# Optimising NK cell metabolism to increase the efficacy of cancer immunotherapy

**DOI:** 10.1186/s13287-021-02377-8

**Published:** 2021-06-05

**Authors:** Chloe Choi, David K. Finlay

**Affiliations:** 1 School of Biochemistry and Immunology, Trinity Biomedical Sciences Institute, Trinity College Dublin, 152-160 Pearse Street, Dublin 2, Ireland; 2 School of Biochemistry and Immunology and School of Pharmacy and Pharmaceutical Sciences, Trinity Biomedical Sciences Institute, Trinity College Dublin, 152-160 Pearse Street, Dublin 2, Ireland

**Keywords:** Natural Killer cells, Tumour microenvironment, Cancer immunotherapy, Metabolism, Nutrients, Mitochondria, Metabolic signalling, mTORC1, cMyc, SREBP, PPAR

## Abstract

Immunotherapy has ushered in an exciting new era for cancer treatment. The recent discovery and success of immune checkpoint blockade and chimeric antigen receptor (CAR) T cell adoptive cell transfer has raised interest in using other immune cells, including Natural Killer (NK) cells, which might overcome some limitations with CAR T cell therapy. In this review article, we discuss the evidence that cellular metabolism is crucial for NK cell effector function. Additionally, potential strategies to optimise the metabolism of therapeutic NK cells for improved function within the metabolically adverse tumour microenvironment will be explored.

## Introduction

The NK cell’s capacity to recognise and rapidly kill tumour cells without damaging healthy tissue suggests its potential for allogenic cancer immunotherapy. Indeed, there has been several efforts in developing clinical grade methods in generating large numbers of NK cells from multiple sources, for example peripheral blood, umbilical cord blood [1, 2], induced pluripotent stem cells (iPSCs) [3, 4] and immortalised NK cell lines [5]. These methods allow manipulation of NK cells to maximise their antitumour potential, including genetic engineering (CAR-NK) or pre-activation of NK cells prior to adoptive transfer therapy [6]. Clinical trials with allogenic NK cell products have shown to be safe without causing graft versus host disease, neurotoxicity or cytokine release syndrome. Moreover, a comparison of CAR-T and CAR-NK cells in a murine model of ovarian cancer demonstrated equal therapeutic effects with less pathology associated with CAR-NK therapy [4]. Therefore, they hold promise as an “off the shelf” therapy that can be administered on demand to multiple patients.

**Fig. 1:**
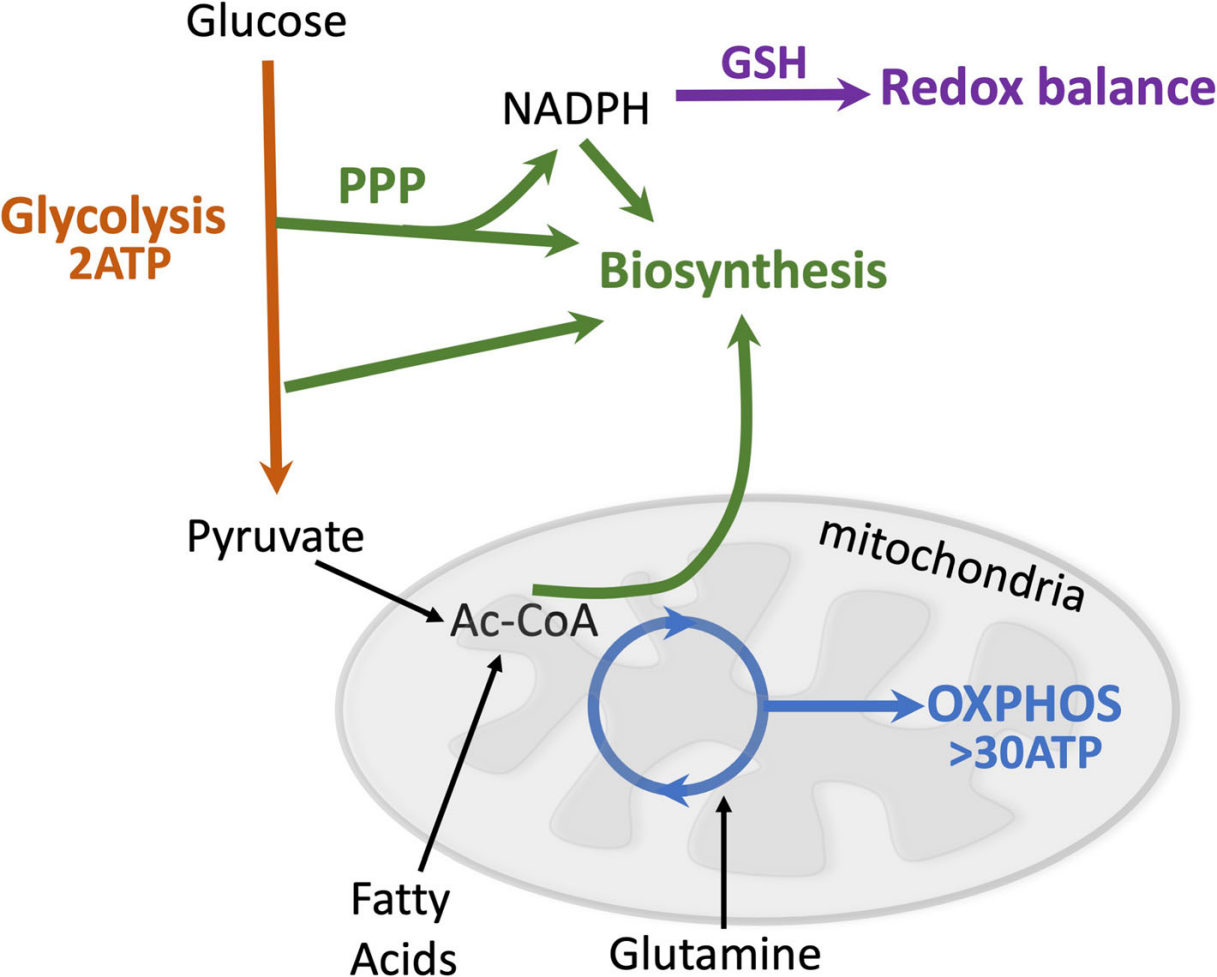
Metabolism supports energy homeostasis and biosynthesis. Cellular metabolism can be configured to efficiently generate energy in the form of adenosine triphosphate (ATP). Glucose is metabolised by glycolysis to pyruvate (generating 2 ATP), which in turn can be used to fuel oxidative phosphorylation (OXPHOS). Pyruvate is converted to Acetyl-CoA in the mitochondria, which fuels metabolic cycles to generate the reducing equivalents NADH and FADH_2_ (not shown) to drive OXPHOS (generating > 30 ATP). Fatty acids are broken down in the mitochondria through a process called β-oxidation that yields acetyl-CoA. Glutamine can also be a fuel for mitochondrial OXPHOS. In addition to fuelling ATP synthesis, glucose and glutamine can also be metabolised and used to support biosynthetic processes. Metabolic intermediates can be diverted into pathways to generate biosynthetic precursors important for the synthesis of lipids, nucleotides and proteins. Glucose can also be diverted into the pentose phosphate pathway (PPP) which is important for nucleotide synthesis and the generation of NADPH. NADPH is an important cofactor for biosynthetic pathways and for the reduction of glutathione, which quenches reactive oxygen species thus supporting redox balance

While NK cell-based immunotherapy is showing good results in blood borne and “hot” tumours like acute myelogenous leukaemia (AML), chronic lymphocytic leukaemia (CLL) and acute lymphoblastic leukaemia (ALL), the efficacy to treat solid or “cold” tumours is often poor. One reason for this is that the immunosuppressive effects of the tumour microenvironment (TME) limit treatment efficacy. Factors contributing to this include tumour-derived cytokines and the hypoxic, acidic and nutrient depleted environment of the TME. It is known that the TME can downregulate NK cell activation directly or indirectly (reviewed elsewhere [7]), and most strategies to modulate NK cells for therapy have focused on cytokine stimulation and the use of monoclonal antibodies against inhibitory NK cell receptors [6]. However, it should also be considered that the TME can influence energy consumption and metabolic reprogramming of immune cells and in doing so limit NK cell anti-tumour responses. Cellular metabolism provides for the energetic and biosynthetic needs of the cell and also supports other important functions such as maintaining redox balance (Fig. 1). In this review, we will discuss how identifying key cellular and molecular mechanisms that regulate NK cell metabolism can provide strategies to overcome the immunosuppressive TME.

## Metabolism supporting NK cell responses

Immune cells have widely varied metabolic signatures to match their diverse functions. Substantial detail about the metabolic configurations of NK cells has emerged in recent years. Murine and human NK cells analysed directly ex vivo have relatively low basal rates of glycolysis and oxidative phosphorylation (OXPHOS) [8–10]. In humans, the two main subsets of NK cells in the blood, CD56^bright^ and CD56^dim^ NK cells, have different metabolic profiles. CD56^bright^ NK cells are the major producers of cytokines including IFN-γ (interferon-γ) and TNF-α (tumour necrosis factor-α), while CD56^dim^ NK cells primed for cytotoxicity with high expression of the cytotoxic machinery. CD56^dim^ NK cells purified directly from human peripheral blood mononuclear cells (PBMC) at steady state have higher rates of glycolysis and OXPHOS and increased mitochondrial mass when compared to CD56^bright^ NK cells [9]. There is also some evidence that human NK cells from blood and tissues such as the spleen and liver have different metabolic profiles [11]. Upon short-term activation (4–6 h) with cytokines (IL12, IL15) or through NK cell receptors (NK1.1 and Ly49D ligation), murine NK cells can exert their effector functions without inducing changes to metabolic rates. However, these low metabolic rates are important for sustaining acute NK responses in terms of IFN-γ production [12]. This suggests that NK cells are equipped with the metabolic machinery required to carry out these immediate innate functions. However, following activation for longer periods, NK cells acquire enhanced effector functions, and this is associated with clear metabolic changes. For example, murine NK cells activated by cytokine for 18 hours undergo robust metabolic changes that are necessary for supporting NK cell cytokine production and cytotoxicity. Various combinations of cytokines upregulate glycolysis and OXPHOS in murine NK cells that are accompanied by increased expression of key nutrient transporters, glycolytic enzymes, increased mitochondrial mass and metabolic fluxes [12–15]. Similarly, in human NK cells isolated from PBMC, 18-h stimulation with cytokines including IL2, IL12+IL15 or IL12+IL15+IL18 results in increased expression of nutrient receptor expression and enhanced glycolytic flux [9, 16]. More recently, studies have shown IL-21 expanded human NK cells have significantly elevated basal and maximal glycolysis but downregulated OXPHOS compared to peripheral blood NK cells. They also have increased levels of the glucose and amino acid transporters SLC2A1, CD98 and CD71. IL-21-mediated metabolic reprogramming of NK cells was accompanied by enhanced IFN-γ and granzyme B production and their ability to kill tumour cells in an ovarian cancer mouse model [17, 18].

## Mitochondrial fitness and NK cell responses

Mitochondrial dysfunction in NK cells and T cells has been a feature of impaired anti-tumour responses in a number of tumour studies. Typically, dysfunctional NK cells and T cells were found to have altered mitochondrial morphology with small individual mitochondria as opposed to elongated mitochondrial networks. These individual mitochondria also showed impaired metabolic outputs and increased production of reactive oxygen species [10, 19, 20]. There are differences in how human CD56^bright^ and CD56^dim^ NK cells modulate mitochondrial functions following cytokine stimulation. While CD56^bright^ NK cells increase mitochondrial mass, mitochondrial polarisation and OXPHOS in response to stimulation, under the same conditions, CD56^dim^ NK cells show decreases in these mitochondrial parameters [9]. These decreases in mitochondrial function are very pronounced in CD56^dim^ NK cells stimulated with IL12+IL15+IL18 [9] but less so in response to other cytokine combinations (unpublished data). Interestingly, interventions that preserve the mitochondrial functions of CD56^dim^ NK cells appear to increase the longevity of these cells [9]. Indeed, these data fit with an emerging narrative that maintaining mitochondrial fitness is an essential metabolic requirement for prolonged lymphocyte responses. The formation of long-lived memory NK cells in the mouse cytomegalovirus model was disrupted in NK cells deficient for mitophagy, a process required for correcting mitochondrial damage [21]. Similarly, mitochondrial function has been found to be important in the formation of long-lived T cell memory CD8+ T cells [22]. Importantly, strategies to improve mitochondrial health through promoting the generation of elongated mitochondrial networks through the inhibition of mitochondrial fission have been found to benefit NK cell anti-tumour responses [19].

## Nutrient transport into NK cells

**Fig. 2:**
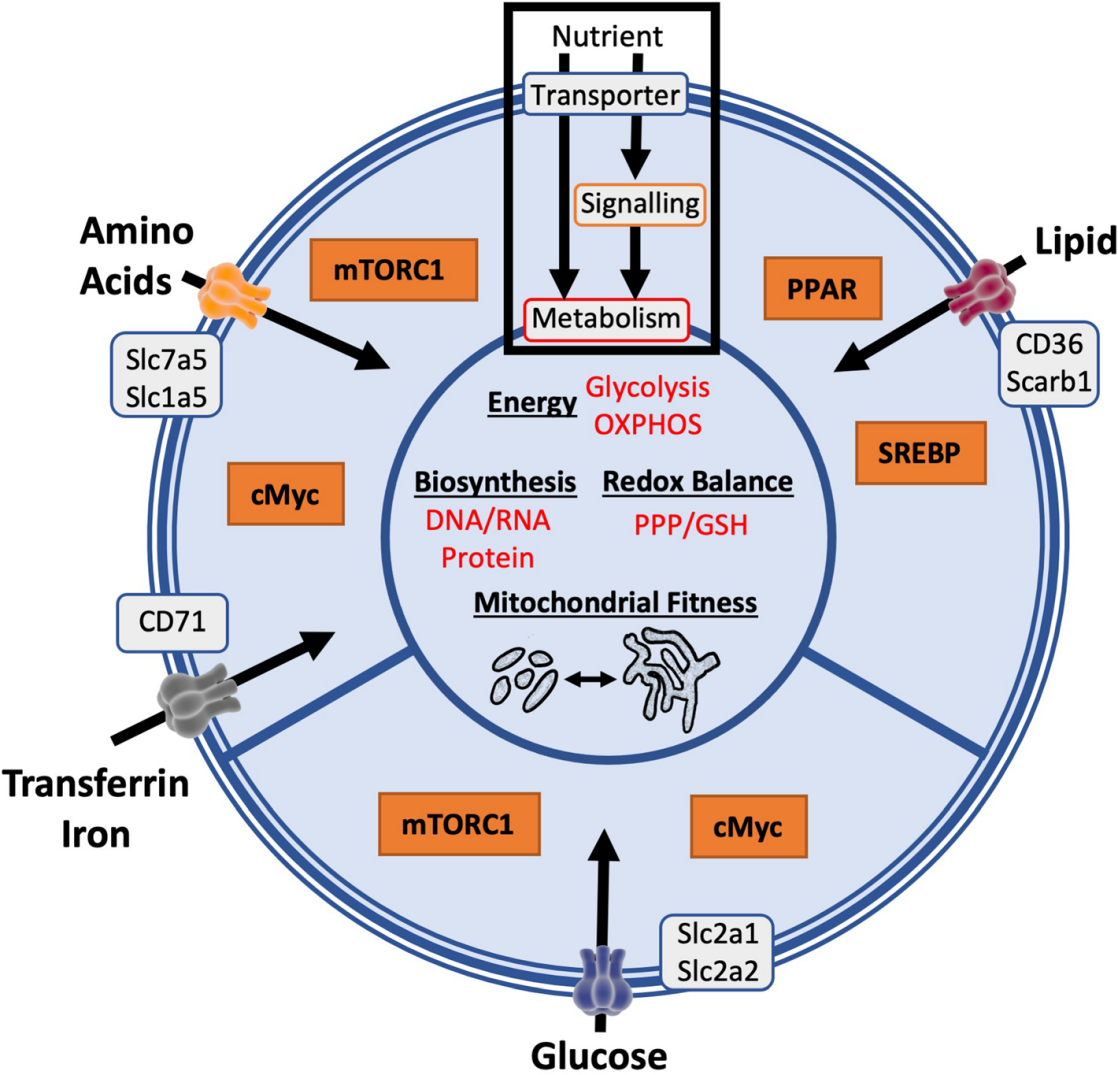
Nutrient transport supports NK cell signalling and metabolism. Numerous nutrient transporters that are important for NK cell metabolism have been identified. Slc2a1 and Slc2a3 (also called Glut1 and Glut3) are the major glucose transporters on NK cells. Glucose supports energy production and pathways for biosynthesis and maintaining redox balance in NK cells. The metabolic regulators mTORC1 and cMyc are sensitive to levels of glucose and can be inactive when NK cells are starved of glucose. The amino acid transporter Slc1a5 (glutamine uptake) and Slc7a5 (large neutral amino acid uptake) are also important for sustaining cMyc and mTORC1 signalling, energy production and biosynthesis. The transferrin receptor CD71 is a cMyc target gene and mediates the uptake of iron that is used as a cofactor for diverse enzymes, notably the complexes of the electron transport chain in the mitochondria. Under certain circumstances scavenging receptors for the uptake of lipids and cholesterol can be expressed on NK cells, including CD36 and Scarb1. Fatty acids can act as agonists for PPAR nuclear receptors and this has been linked to metabolic dysfunction, including decreased mitochondrial fitness, in obesity. Cholesterol and oxidised cholesterol molecules such as 25-hydroxycholesterol inhibit SREBP activation causing profound metabolic and functional abnormalities

The transport of nutrients into murine and human NK cells is essential to support increased metabolic rates, which is mediated by increased expression of nutrient tranporters for glucose (Slc2a1), amino acids (Slc7a5, Slc3a2, Slc1a5) and iron-bearing transferrin (CD71, the transferrin receptor) (Fig. 2) [8, 14, 16, 23]. Cytokine-stimulated CD56^bright^ NK cells can also upregulate the expression of transporters for fatty acids, such as CD36, though the significance of this with respect to the function of this NK cell subset is not clear [9]. Whether fatty acids are a fuel source for NK cells is unclear and requires further investigation. Limiting the uptake of glucose or certain amino acids negatively impacts upon NK cell effector functions through the direct inhibition of cellular metabolism and through inhibition of signal transduction pathways including the mammalian target of rapamycin complex 1 (mTORC1) and cMyc [8, 9, 14–16, 24]. In contrast, the excessive uptake of fatty acids is associated with NK cell dysfunction in some pathological situations, circumstances where transporters of lipids are highly upregulated [25, 26]. For instance, NK cells are highly dysfunctional in obese humans and this is linked to lipid loading of the NK cells and the aberrant activation of peroxisome proliferator-activated receptors (PPAR) signalling [25, 27]. In a mouse model of B cell lymphoma, dysfunctional NK cells expressed the lipid scavenging receptors CD36 and Scavenger Receptor Class B Member 1 (Scarb1) had elevated lipid droplet content [26].

## How the TME can affect NK cell metabolism

Changes in cellular metabolism are integrally linked to the functional fate of immune cells [28, 29]. Limiting glycolysis or OXPHOS in human or murine NK cells results in impaired IFN-γ and Granzyme B production and cytotoxicity [15, 16]. Inhibition of amino acid transporters such as Slc1a5 and Slc7a5/CD98 reduces IFN-γ production and cytotoxicity [8, 23]. In pathological settings like the TME, limited nutrient availability may restrict NK cell metabolism thus impairing NK cell function [30–32]. Furthermore, tumours can increase lipid metabolism and enrich the TME with lipids to facilitate tumour progression, including proliferation, migration and evasion of the immune response (reviewed elsewhere [33, 34]). Accumulation of excess intracellular lipids in NK cells result increased PPAR-γ/PPAR-δ signalling and has profound inhibitory effects on metabolic activity and NK cell cytotoxicity [25, 26]. Other factors in the TME have been reported to inhibit NK cell metabolism. For instance, transforming growth factor beta (TGF-β) which is elevated in metastatic breast cancer patients, can directly suppresses NK cell metabolism through both mTORC1 inhibition and mTORC1-independent inhibition of mitochondrial metabolism [35–37]. Acidification of the TME can also negatively effect NK cells. Increased acidification in the TME in human colorectal liver metastasis leads to intracellular acidification of NK cells, mitochondrial stress and ultimately NK cell apoptosis [38]. In the complex TME, there are additional metabolites that can dampen NK cell activity include oxysterols, lactic acid, L-Kynurenine, prostaglandin E_2_ and adenosine [39–44]. In breast cancer patients, tumour infiltrating NK cells upregulate CD73, the ectoenzyme that metabolise adenosine monophosphate to adenosine [45]. Thus, unravelling the interconnection between cancer and immune metabolism will reveal targets for future immunotherapies.

## Importance of metabolic configuration

Activated NK cells metabolise glucose primarily through aerobic glycolysis to pyruvate and then lactate. Interesting, while glucose is also the main fuel for supporting OXPHOS, this is not through fuelling the tricarboxylic acid (TCA) cycle. Instead, activated NK cells metabolise pyruvate though the citrate malate shuttle, a cycle that spans the mitochondrial membrane [14, 28]. In contrast, glutamine and glutaminolysis do not act to fuel OXPHOS in activated NK cells [8]. This is an interesting metabolic configuration that is maintained by the Sterol regulatory element binding protein (SREBP) transcription factors that control the expression of key components of this citrate-malate-shuttle, ATP-citrate lyase and Slc25a1 [14]. Therefore, SREBP is important for sustaining elevated rates of glycolysis and OXPHOS in NK cells [14]. This metabolic configuration has important implications for NK cell anti-tumour responses because the activation of SREBP transcription factors is potently inhibited by cholesterol and oxysterols. Elevated cholesterol metabolism with enriched cholesterol derivatives in the TME have profound effects on the immune response (reviewed elsewhere [34, 44]). For instance, colon cancer and myeloma patients have high cholesterol content which contributed to CD8+ T cell exhaustion and impaired anti-tumour function [46]. Naturally occurring inhibitors of SREBP, including 27-hydroxycholesterol and 25-hydroxycholesterol, may be increased in the TME and are found to be elevated in the circulation of patients with breast, gastric and colorectal cancers [47–50]. Inhibition of SREBP impairs cytokine production and cytotoxicity of both murine and human NK cells in vitro and curtails anti-tumour responses in an adoptive NK cell mouse model [14].

## Metabolic signalling as potential therapeutic targets in NK cells

As discussed above, the transcription factor SREBP has a key role in facilitating elevated cellular metabolism in cytokine activated NK cells. There are a number of other signalling molecules that have been identified to be important for NK metabolic responses that are discussed below.

*mTORC1* is a master regulator of cellular metabolism that is important both for NK cell development and the responses of activated NK cells [13]. Cytokine stimulation of murine and human NK cells result in robust mTORC1 activation, which is required for metabolic and functional responses [13, 15, 16]. With this in mind, there have been efforts towards engineering NK cells to maintain mTORC1 activity to improve anti-tumour response and NK cell persistence. For example, silencing of the intracellular immune checkpoint cytokine-inducible SH2-containing protein (CIS; encoded by the gene CISH) in murine and human NK cells increased their sensitivity to IL15 resulting in increased single cell polyfunctionality, persistence in vivo and enhanced anti-tumour responses. Deletion of CISH in iPSC-derived NK [51] or cord blood-derived NK [52] resulted in increased JAK/STAT signalling and mTORC1 signalling leading to increasing NK cell metabolic fitness that directly contributed to improved anti-tumour responses [51, 53]. While studies highlight the positive roles of mTORC1 for NK cell responses, it is interesting to note constant exposure to IL15, which is known to stimulate mTORC1 signalling, can result NK cell exhaustion and reduced cytotoxicity [54].

*cMyc* regulates the expression of the metabolic machinery required to support elevated rates of glycolysis and OXPHOS [8]. Upon cytokine stimulation, cMyc deficient NK cells have an altered metabolic phenotype including reduced glycolytic enzyme expression, reduced mitochondrial mass and impaired functional responses. It is worth noting that while in many cell types cMyc is found to be regulated downstream of mTORC1, this is not necessarily the case in NK cells [8] or indeed in effector CD8+ T cells [55, 56]. Sustained cMyc protein expression in cytokine stimulated NK cells is completely independent of mTORC1 activity [8]. However, cMyc protein expression is acutely sensitive to the supply of amino acid through amino acid transporters, most notably Slc7a5, as it sustains high rates of cMyc synthesis to offset continuous cMyc protein degradation. Inhibition of amino acid transport through Slc7a5 or amino acid withdrawal, as might be the case in the TME, results in the rapid loss of cMyc expression and attenuated NK cell responses [8]. cMyc protein expression is particularly sensitive to the levels of glutamine; depriving NK cells of glutamine alone was sufficient to cause the loss of cMyc protein. The activity of SREBP is also required for maximal cMyc protein expression; the SREBP inhibitor 25-hydroxycholesterol substantially reduces cMyc levels in cytokine stimulated murine NK cells [57].

cMyc signalling can also be regulated by stress response pathways. Inositol-requiring enzyme 1 (IRE1α) responds to endoplasmic reticulum stress and activates X-box-binding protein 1 (XBP1). Cytokine stimulation of NK cells induces XBP1 transcription factor activation to promote NK proliferation, survival and optimal function [58, 59]. IRE1α and XBP1 are important for optimal mitochondrial fitness through regulation of cMyc [58]. Mice lacking the expression of IRE1α or XBP1 in NK cells had increased tumour burden using the B16 melanoma tumour model. Increased tumour growth was associated with reduced cMyc expression in NK cells, reduced NK cell proliferation and absolute numbers of tumour infiltrating NK cells [58].

Strategies to stabilise cMyc in tumour infiltrating NK cells might provide a metabolic advantage to these cells through sustaining glucose metabolism and mitogenesis. For instance, the kinase glycogen synthase kinase-3 (GSK3) can promote cMyc degradation. Murine NK cells placed in conditions of amino acid deficiency, conditions that destabilise cMyc, showed increased cMyc expression in the presence of a GSK3 inhibitor [8]. GSK3 is overexpressed in NK cells from AML patients and inhibitors of GSK3 restores cytotoxicity in these NK cells. Inhibition of GSK3 also enhanced TNF-α and IFN-γ production and elevated cytotoxicity in NK cells an adoptive transfer mouse models of AML and ovarian cancer [60, 61]. In fact, GSK3 inhibitors are currently tested in clinical trials for their anti-tumour activity (NCT01632306) (NCT01287520) (NCT01214603).

While glutamine is not a key fuel for NK cell metabolism, it is an important metabolite to sustain cMyc protein expression. Targeting glutamine metabolism within tumours could be another route to supporting cMyc expression in tumour infiltrating NK cells. The compound JHU083 inhibits a broad range of glutamine requiring enzymes specifically within the tumour and has been shown to decrease tumour growth and improve survival by dismantling the immunosuppressive TME and enhancing T cell function [62]. This broad suppression of glutamine metabolism was accompanied by an increased in glutamine availability that could support cMyc protein expression in NK cells. JHU083 treatment also led to increased glucose availability and a decrease in tumour hypoxia and acidity. Increased glucose would be beneficial through supporting NK cells glycolytic metabolism. Decreased acidification of the TME would support NK cell survival, as NK cells are susceptible to apoptosis in low pH TME [38]. Therefore, blocking general glutamine metabolism within tumours may be an approach to support NK cell anti-tumour responses.

*Hypoxia-inducible factor 1α (HIF1α)*. Most solid tumours show high oxygen consumption and disorganised vascularisation generating an immunosuppressive hypoxic environment (reviewed elsewhere [63, 64]). One way that cells adapt to hypoxia is upregulating the protein expression of HIF1α, an important metabolic regulator that supports cellular glycolysis. HIF1α also is an important metabolic regulator under certain normoxic conditions and plays an crucial role in supporting glycolysis and effector function in CD8+ cytotoxic T cells [55]. However, HIF1α is not a key regulator of NK cell metabolism and the data suggests that HIF1α expression in NK cells can have pro-tumour effects [65, 66]. HIF1α-deficient NK cells have normal metabolic and effector function responses following IL2+IL12 cytokine stimulation under normoxic conditions, suggesting HIF1α is dispensable for normal cytokine-induced NK cell responses [8]. In solid tumours, prolonged exposure to hypoxia induces HIF1α expression in tumour infiltrating NK cells and negatively regulates IL18-dependent NFκB activity [67]. In this study, HIF1α-deficient NK cells are more responsive to IL18 and hindered growth of RMA-S and LLC tumour models, showing enhanced OXPHOS and NFκB activity which correlated with increased expression of IFN-γ and Granzymes [67]. In a different study, the deletion of HIF1α was also shown to be beneficial, albeit through a different mechanism. Loss of HIF1α in NK cells inhibited tumour growth despite attenuated cytotoxicity. Instead, HIF1α deficient NK cells increased the concentration of an angiogenic cytokine, vascular endothelial growth factor (VEGF) and disabled productive angiogenesis required for tumour progression [68]. HIF1α regulation in NK cells is complex and requires further investigation to understand its potential in cancer therapy.

## Additional ways metabolism may affect NK cell antitumour functions

The infiltration of NK cells into tumours has been shown to be linked to a good prognosis in a number of cancer types [69–72]. Two of the key factors that are required for tumour infiltration of NK cells are IFN-γ and the chemokine receptor CXCR3 [73]. While there is an abundance of evidence that the production of IFN-γ by lymphocytes is affected by cellular metabolism, whether cellular metabolism in NK cells affects the expression of the CXCR3 has not been studied. In CD8 T cells, the transcription factor HIF1α acts as a negative regulator of multiple chemokine receptors including CXCR3, which might suggest that tumour hypoxia could affect the tumour infiltration of cytotoxic lymphocytes [55]. Indeed, mice specifically lacking HIF1α within the NK cell compartment have increased NK cell anti-tumour responses and reduced tumour growth [67]. Hypoxia has been linked to altered expression of chemokine receptors on human NK cells resulting in the sustained expression of the lymph node homing CCR7 [65]. The cytokine TGF-β inhibits mitochondrial metabolism in human NK cells in the context of breast cancer and in CD8+ T cell suppresses CXCR3 expression and tumour homing [74]. However, further studies are required to understand the how and to what extent the TME and metabolic conditions therein impact upon the infiltration of NK cells into tumours.

Beyond direct cytotoxicity towards tumour cells, NK cells have indirect roles in mediating the immune response in the TME. NK cells can secrete multiple cytokines, growth factors and chemokines that can influence the activity of other immune cells. For instance, a recent study show NK cells increasing the abundance of conventional type I DC in tumour sites through secreting chemoattractant CCL5 and XCL1/2, and the extent of this process correlates with patient survival in several cancer types [42]. Additionally, NK cells are a source of Fms-related tyrosine kinase 3 ligand (FLT3L), a key cytokine for DCs and their frequency in tumour sites directly correlate with survival in patients with melanoma receiving anti-PD-1 [75]. Therefore, it would be very interesting to address how NK cell metabolism affect these other aspects of NK cell function.

The complex TME also impacts upon the activating and inhibitory receptors expressed by infiltrating NK cells and thus contribute to NK cell dysfunction. A number of different cell types in the TME, such as regulatory T cells, myeloid dervived suppressor cells and fibroblasts, and the immunosuppressive factors they produce are likely to contribute to altered NK cell receptor expression [76]. There is much interest in targeting inhibitory NK cell receptors such as T cell immunoreceptor with Ig and ITIM domains (TIGIT) and NKG2A for cancer immunotherapy [77, 78]. However, little is known is known about whether the adverse metabolic conditions within the TME contribute to the increased expression of these inhibitory NK receptors on tumour infiltrating NK cells.

## Concluding remarks

**Fig. 3:**
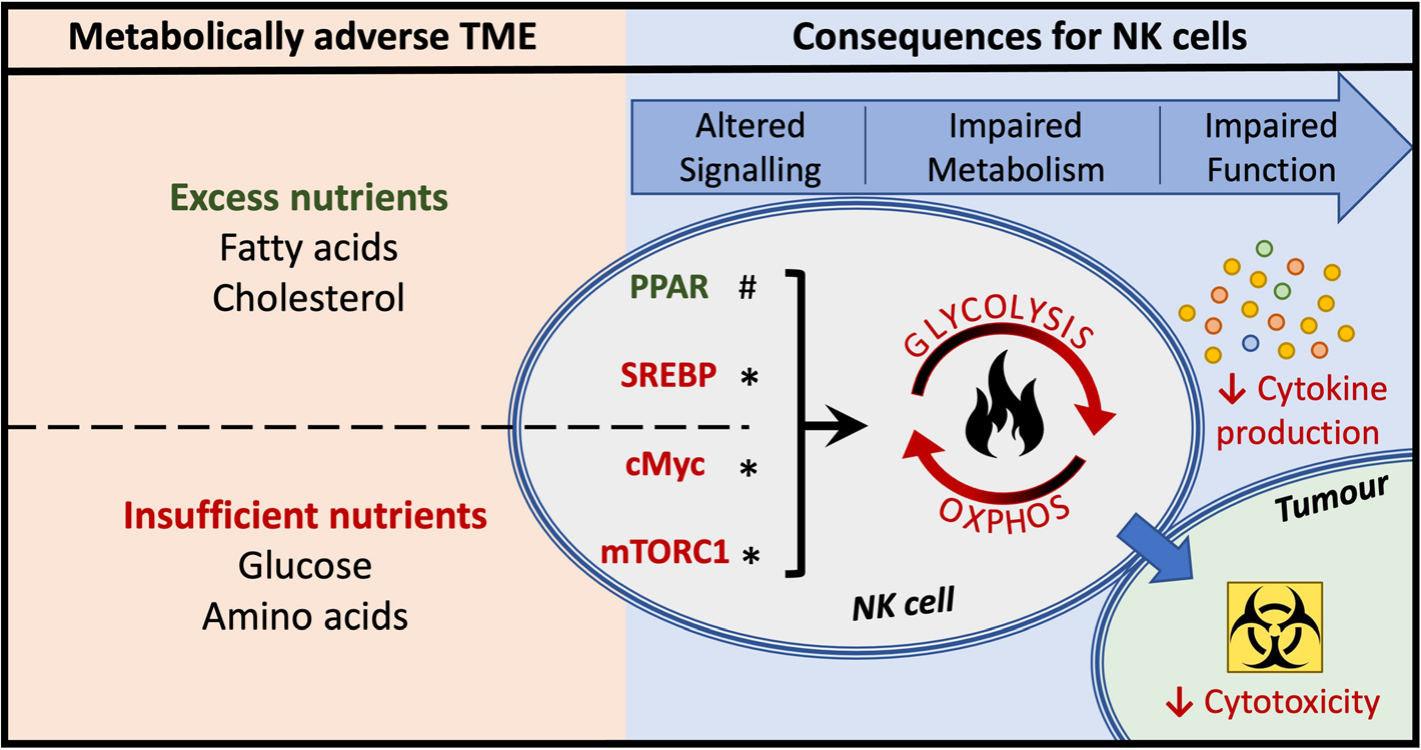
Targeting signalling to support NK metabolism in the TME. Metabolically adverse TME can be the result of excess nutrients such as fatty acids and cholesterol/oxysterols that can lead to the activation of PPAR and the inhibition of SREBP in NK cells, respectively. Other factors in the TME including tumour growth factor beta (TGF-β), pH, lactate has been shown to interfere with metabolic signalling in NK cells (not shown). Insufficient nutrients, including glucose and amino acids, will inhibit the activity nutrient sensors such as cMyc and mTORC1 in NK cells. Such alterations in NK signalling negatively affect NK cell metabolism, including flux through glycolysis and OXPHOS and in doing so impair NK cell anti-tumour responses. Strategies to improving NK cell function within the TME could involve boosting (*) or inhibiting (#) the relevant signalling pathway corresponding to the metabolic conditions within the TME

Despite the remarkable responses of a subset of patients to cancer immunotherapy, many patients remain resistant to these therapies. Many NK cell-based immunotherapies focus on promoting NK cell cytotoxicity. For instance, monoclonal antibodies blocking inhibitory NK cell receptor interaction with their ligands on tumour cells or promoting activatory NK cell receptor signalling. Other therapies include adoptive cell transfer of cytokine preactivated NK cells or CAR NK cells. A major challenge with these therapies is the eventual development of NK cell exhaustion. Therefore, the metabolically challenging TME that these immunotherapeutic products are subjected to must not be overlooked. Identifying key cellular and molecular mechanisms that regulate NK cell metabolism will reveal new and exciting strategies to engineer innovative CAR NK cells to overcome the immunosuppressive TME and promote longevity and metabolic and functional fitness (Fig. 3).

## Data Availability

Not applicable.

## Abbreviations

CAR: Chimeric antigen receptor
NK: Natural Killer
iPSCs: Induced pluripotent stem cells
AML: Acute myelogenous leukaemia
CLL: Chronic lymphocytic leukaemia
ALL: Acute lymphoblastic leukaemia
TME: Tumour microenvironment
OXPHOS: Oxidative phosphorylation
IFN-γ: Interferon-γ
TNF-α: Tumour necrosis factor-α
PBMC: Peripheral blood mononuclear cells
mTORC1: Mammalian target of rapamycin complex 1
PPAR: Peroxisome proliferator-activated receptors
Scarb1: Scavenger Receptor Class B Member 1
TGF-β: Transforming growth factor beta
TCA: Tricarboxylic acid
SREBP: Sterol regulatory element binding protein
CISH: Cytokine-inducible SH2-containing protein
IRE1α: Inositol-requiring enzyme 1
XBP1: X-box-binding protein 1
GSK3: Glycogen synthase kinase-3
HIF1α: Hypoxia-inducible factor 1α
VEGF: Vascular endothelial growth factor
TIGIT: T cell immunoreceptor with Ig and ITIM domains
ATP: Adenosine triphosphate
PPP: Pentose phosphate pathway
